# Adolescent substance use behavior and suicidal behavior for boys and girls: a cross-sectional study by latent analysis approach

**DOI:** 10.1186/s12888-017-1546-1

**Published:** 2017-12-08

**Authors:** Peng-Wei Wang, Cheng-Fang Yen

**Affiliations:** 1 Department of Psychiatry, Kaohsiung Medical University Hospital, 100 Tzyou 1st Road, Kaohsiung, 807 Taiwan; 2 Department of Psychiatry, Faculty of Medicine and Graduate Institute of Medicine, College of Medicine, Kaohsiung Medical University, Kaohsiung, Taiwan

## Abstract

**Background:**

Adolescent suicidal behavior may consist of different symptoms, including suicidal ideation, suicidal planning and suicidal attempts. Adolescent substance use behavior may contribute to adolescent suicidal behavior. However, research on the relationships between specific substance use and individual suicidal behavior is insufficient, as adolescents may not use only one substance or develop only one facet of suicidal behavior. Latent variables permit us to describe the relationships between clusters of related behaviors more accurately than studying the relationships between specific behaviors. Thus, the aim of this study was to explore how adolescent substance use behavior contributes to suicidal behavior using latent variables representing adolescent suicidal and substance use behaviors.

**Method:**

A total of 13,985 adolescents were recruited using a stratified random sampling strategy. The participants indicated whether they had experienced suicidal ideation, planning and attempts and reported their cigarette, alcohol, ketamine and MDMA use during the past year. Latent analysis was used to examine the relationship between substance use and suicidal behavior.

**Results:**

Adolescents who used any one of the above substances exhibited more suicidal behavior. The results of latent variables analysis revealed that adolescent substance use contributed to suicidal behavior and that boys exhibited more severe substance use behavior than girls. However, there was no gender difference in the association between substance use and suicidal behavior.

**Conclusion:**

Substance use behavior in adolescents is related to more suicidal behavior. In addition, the contribution of substance use to suicidal behavior does not differ between genders.

## Background

Suicidal behavior is a broad term consisting of a broad scope of thoughts and actions, with at least an intent to result in death, ranging from ideation to active, self-inflicted death [1]. Previous studies have indicated that suicidal ideation is associated with suicidal planning and attempts and that suicidal planning contributes to the occurrence of suicide attempts [2, 3]. It is particularly difficult to differentiate suicidal ideation from suicidal attempts among adolescents [4]. Furthermore, studies have also demonstrated that suicidal ideation, planning and attempts may result from some candidate genes or imbalances in the interactions among genes [5, 6], indicating that suicidal ideation, planning and attempts are interrelated and that suicidal behavior can be viewed as a cluster of related phenomena which result from an unobserved force.

Adolescence is a critical period regarding the initial use of tobacco, alcohol and other substances [7], and the prevalence of adolescent substance use is high in many countries. In Australia, the rate of current tobacco use in adolescents is 10%, and the rate of illicit drug during the past 12 months is 16% [8]. In the United States, 14% of grade 12 adolescents have used tobacco, and the lifetime prevalence of adolescent cannabis use is 23% [9]. A previous study in Taiwan reported that the overall prevalence of any substance use disorder is 11%, with tobacco being the most common [10]. Research has found that the use of one type of substance may contribute to the use of another type of substance among adolescents [11, 12]. In addition, adolescents who use different types of substances may share similar genetic and environmental factors [13, 14]. Therefore, adolescent substance use can also be viewed as a class of observable behaviors which result from an unobserved force.

Adolescent substance use may result in many health-related problems, such as depression and abnormal brain functioning [15, 16]. Substance use is also one of the risk factors that contribute to adolescent suicidal behavior [17]. Previous studies examining the relationship between substance use and suicidal behavior among adolescents have focused primarily on the types of substance use that increase the risk of suicidal behavior and on the degrees of association between the use of various types of substances and suicidal behavior [18, 19]. However, as described above, substance use can be considered a cluster of related behavioral phenomena. In addition, poly-substance use is common in adolescents [20], and research has demonstrated that combined use of multiple substances increases the risk of suicidal behavior [21]. Further research on the relationship between substance use and suicidal behavior in adolescents should examine multiple domains of substance and suicidal behavioral phenomena because both have a cluster of related behavioral phenomena. A latent variable can represent the unobserved force to result in several related observable behaviors. Latent variables analysis can examine the relationships between observable behavior and the unobserved force. Furthermore, latent analysis can describe the relationships among a class of events or observable phenomena that share similar characteristics rather than making concrete statements restricted to the relationships between more specific variables [22]. Therefore, latent variables analysis may be a good method by which to further our understanding of the relationship between substance use and suicidal behavior in adolescents.

Research has demonstrated that girls are more likely to experience suicidal ideation and attempts than boys [23] and that boys are more likely to use illicit substances, tobacco and alcohol than girls [24]. However, the gender influence on the relationship between substance use and suicidal behavior in adolescents is unclear. The present study used latent variable to represent two unobserved forces for suicidal and substance user behavior. Then we examined the relationship between two latent variables. The study aimed to examine the relationships between the use of four substances commonly used by adolescents (cigarettes, alcohol, ketamine, and 3,4-methylenedioxymethamphetamine [MDMA]) and three phenomena of suicidal behavior (suicidal ideation, planning and attempts) in adolescents. The present study also explored the gender difference in the contribution of adolescent substance use to suicidal behavior. The hypotheses were 1) adolescent substance use contributes to adolescent suicidal behavior in both boys and girls and 2) a gender difference exists in the strength of the contribution of adolescent substance use to suicidal behavior.

## Method

### Participants

The current study was based on data from the 2004 Project for the Health of Adolescents in Southern Taiwan, a research program studying the mental health status of adolescents living in four counties and three metropolitan areas in southern Taiwan. On the basis of the Taiwan-Fukien Demographic Fact Book [25], 10 senior high/vocational schools and 11 junior high schools were randomly selected from non-urban areas, and 19 senior high/vocational schools and 12 junior high schools were randomly selected from urban areas. The classes in the selected schools were stratified into three levels according to grade. A total of 17,130 were then randomly selected and 13,985 adolescents (81.64%) completed all research questionnaires. The final sample size of each stratum was proportionate to the population size of the strata based on districts, schools and grades. The mean age of this group was 14.75 years (standard deviation [SD]: 1.77 years, range: 12-19 years). The protocol was approved by the Institutional Review Board of Kaohsiung Medical University.

### Assessment

#### The questionnaire for experience in substance use (Q-ESU)

The Q-ESU was used to assess each participant’s experience of smoking, alcohol consumption, and illicit drug use (including ketamine and MDMA) during the preceding year because these were the most popular substances for adolescents [26]. In this study, the responses of the participants were dichotomously divided to represent whether the participants had smoked cigarettes at least once every week, consumed alcohol at least once every week, and had ever used ketamine and MDMA during the preceding year. The 2-week test-retest reliability of the items in the pilot study (*k*) was 0.704-0.763 (*P* < 0.001). The *k* coefficients of agreement between the information provided by the participants and reports from their parents regarding smoking, alcohol consumption, and illicit drug use ranged from 0.602 to 0.852 (*P* < 0.01).

#### Suicidal ideation, planning, and attempts

The 5-item questionnaire which were adapted from the epidemiological version of the Kiddie Schedule for Affective Disorders and Schizophrenia (K-SADS-E) [27] was used to assess the occurrence of three forms of suicidal ideation (items 1 to 3), suicidal planning (item 4) and suicide attempts (item 5) in the past year [28]. Each question elicited a “yes” or “no” answer. The Cohen’s kappa coefficient of agreement (κ) between the participants’ self-reported suicide attempts and their parents’ reports was 0.541 (*P* < 0.001) [28]. In this study, the participants who answered “yes” to any of the first three items were classified as having suicidal ideation, those who answered “yes” to the fourth item were classified as having made suicidal plans, and those who answered “yes” to the last item were classified as having attempted suicide.

### Procedure and statistical analysis

Research assistants explained the purpose of the study to all students in the classes, emphasized respect for the students’ privacy, and encouraged the students to participate. The adolescents were asked to anonymously complete the questionnaire based on the explanations of the research assistants and under the direction of the research assistants. Each student received a gift that was worth NT$33 (one US dollar) at the end of the assessment.

**Fig. 1:**
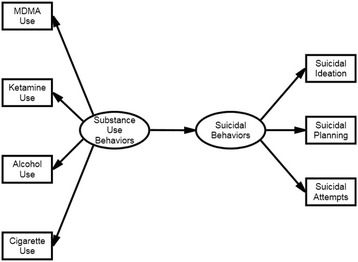
Model of the relationship between adolescent substance use and suicidal behavior

The descriptive data analysis was performed using IBM SPSS Statistics 20. The prevalence rates of the use of each substance in the preceding year and the proportions of adolescents with different phenomena of suicidal behavior were examined. We compared the rates of substance use and suicidal behavior in boys and girls using the Chi-square test. The rates of suicidal behavior in substance users and non-substance users in both genders were also analyzed using the Chi-square test. A measurement invariance (MI) test enabled model comparison with latent variables [29]. We used MI to demonstrate identical constructs with the same structure across the genders in our model, in which substance use and suicidal behavior served as latent variables. After determining the measurement invariance, we compared the mean level of substance use and the strength of the contribution of substance use to suicidal behavior between boys and girls. For these analyses, multilevel structural equation modeling (SEM), which provided a framework for examining the measurement invariance and the differences in latent variables between groups, was performed using STATA 15 [30]. The multilevel SEM can adjust the clustering of students in classrooms and schools as well as analyze categorical data by using cluster robust maximum likelihood [31, 32]. The cluster robust maximum likelihood bases on Taylor series linearization approach for cluster and categorical data [33]. Although, our sample was proportionate to the population size of the strata based on districts, schools and grades, we used age as weighting adjustment during analysis to draw a more reliable result. The hypothesized model is presented in Fig. 1. The indices used to estimate the goodness of fit of the model were the comparative fit index (CFI) >0.9, and the root mean square error of approximation (RMSEA) <0.1. The index used to estimate the goodness of fit of the model and measurement invariance was Standardized root mean squared residual (SRMR) [34, 35]. A two-tailed *P* value of less than 0.05 was considered statistically significant.

## Results

The rate of smoking once or more during the past month was 6.76% which was closed to our national tobacco survey [36]. The rate of alcohol use once or more during the past month was 17.41% which was closed to the previous result [37]. The rate of ketamine use during past year was also closed to results from a national survey study [26]. The results of the comparisons of substance use and suicidal behavior between girls and boys are displayed in Table 1. The results indicated that girls had lower rates of smoking and alcohol use than boys; however, no significant gender differences in the rates of MDMA and ketamine use were found. Girls had higher rates of suicidal ideation, planning and attempts than boys.

**Table 1:** Comparisons of substance use and suicidal behavior between girls and boys

	Girls (*n* = 7374) *n* (%)	Boys (*n* = 6611) *n* (%)	*χ* ^2^	*P*
Cigarette use	121(1.64)	311(4.70)	109.27	<0.001
Alcohol use	54(0.73)	112(1.69)	27.50	<0.001
MDMA use	31(0.42)	29(0.44)	0.03	0.898
Ketamine use	28(0.38)	23(0.35)	0.10	0.779
Suicidal ideation	1431(19.41)	800(12.10)	138.74	<0.001
Suicidal planning	921(12.49)	622(9.41)	33.76	<0.001
Suicidal attempts	759(10.29)	358(5.42)	112.85	<0.001

The results of the comparisons of suicidal ideation, planning and attempts between boys and girls with and without substance use are presented in Table 2. Regardless of the type of substance boys used, those with substance use had higher rates of suicidal ideation, planning, and attempts than those without substance use. In addition, girls with any substance use had higher rates of suicidal ideation, planning, and attempts than girls without substance use.

**Table 2:** Rates of suicidal ideation, planning and attempts among boys and girls with and without substance use

	Suicidal ideation *n* (%)	*P* value	Suicidal plaining *n* (%)	*P* value	Suicidal attempts *n* (%)	*P* value
Boys						
Cigarette use						
Yes(*n* = 311)	64 (20.58)	<0.001	60 (19.29)	<0.001	39 (12.54)	<0.001
No(*n* = 6300)	736 (11.68)		562 (8.92)		319 (5.06)	
Alcohol use						
Yes (*n* = 112)	30 (26.79)	<0.001	25 (22.32)	<0.001	19 (16.96)	<0.001
No (*n* = 6499)	770 (11.85)		597 (9.19)		339 (5.22)	
Ketamine use						
Yes (*n* = 23)	10 (38.46)	0.001	8 (34.78)	0.002	5 (21.74)	0.012
No (*n* = 6588)	790 (12.00)		614 (9.32)		353 (5.36)	
MDMA use						
Yes (*n* = 29)	16 (44.44)	<0.001	12 (33.33)	<0.001	9 (25.00)	<0.001
No (*n* = 6582)	784 (11.92)		610 (9.28)		349 (5.31)	
Girls						
Cigarette use						
Yes(*n* = 121)	63 (52.06)	<0.001	49 (40.50)	<0.001	47 (38.84)	<0.001
No(*n* = 7253)	1378 (18.90)		872 (12.05)		712 (9.82)	
Alcohol use						
Yes (*n* = 54)	26 (48.15)	<0.001	23 (42.59)	<0.001	23 (42.59)	<0.001
No (*n* = 7320)	1405 (19.19)		898 (12.27)		736 (10.05)	
Ketamine use						
Yes (*n* = 28)	13 (46.43)	<0.001	10 (33.33)	0.003	9 (47.37)	0.002
No (*n* = 7346)	1418 (19.30)		913 (12.43)		750 (10.21)	
MDMA use						
Yes (*n* = 31)	12 (34.48)	0.027	10 (28.57)	0.009	9 (25.71)	0.007
No (*n* = 7343)	1419 (19.34)		911 (12.41)		750 (10.21)	

The results of the comparisons of suicidal ideation, planning and attempts between boys and girls with substance use are displayed in Table 3. Girls with smoking and alcohol use had higher rates of all types of suicidal behavior than boys with smoking and alcohol use. However, there was no gender difference in any type of suicidal behavior among adolescents with ketamine or MDMA use.

**Table 3:** Comparisons of suicidal ideation, planning and attempts between girls and boys with substance use

	Cigarette use			Alcohol use			Ketamine use			MDMA use		
	Girls (*n* = 121) *n* (%)	Boys (*n* = 311) *n* (%)	*P* value	Girls (*n* = 54) *n* (%)	Boys (*n* = 112) *n* (%)	*P* value	Girls (*n* = 28) *n* (%)	Boys (*n* = 23) *n* (%)	*P* value	Girls (*n* = 31) *n* (%)	Boys (*n* = 29) *n* (%)	*P* value
Suicidal ideation	63 (52.06)	64 (20.58)	<0.001	26 (48.15)	30 (26.79)	0.006	13 (46.43)	10 (38.46)	0.712	12 (34.29)	16 (44.44)	0.264
Suicidal planning	49 (40.50)	60 (19.29)	<0.001	23 (42.59)	25 (22.32)	0.007	10 (33.33)	8 (34.78)	0.838	10 (28.57)	12 (33.33)	0.664
Suicidal attempts	47 (38.84)	39 (12.54)	<0.001	23 (42.59)	19 (16.96)	<0.001	9 (47.37)	5 (21.74)	0.353	9 (25.71)	9 (25.00)	0.945

The SRMR for the unconstrained model、equal factor loading model and equal factor loading factor and intercept model are 0.0093, 0.0093 and 0.0096, respectively. The fit indices for measurement invariance were acceptable (Table 4). This indicated that the model fulfilled measurement invariance and was valid for boys and girls.

**Table 4:** Fitting indices for each model

	CFI	RMSEA	SRMR
Unconstrained model	0.997	0.010	0.0093
Equal factor loading model	0.997	0.011	0.0093
Equal factor loading and intercept model	0.995	0.013	0.0096

Substance use contributed to suicidal behavior in both girls and boys (girls’ structural coefficient: 0.34, *P* < 0.001; boys’ structural coefficient: 0.53, *P* < 0.001; girls’ standardized structural coefficient: 0.18; boys’ standardized structural coefficient: 0.24). However, there was no gender difference in the relationship between substance use behavior and suicidal behavior (Chi-square for differences between parameters: 1.069, *P* > 0.05). In addition, the level of substance use behavior differed significantly between genders (girls’ mean: 0.015; boys’ mean: 0.041; *P* < 0.001) (Table 5).

**Table 5:** Factor loadings, structural coefficient and standardized loading for the structural equation model

			Boys			Girls		
			Factor loadings^a^/ structure coefficient^b^	Standardized Factor loadings^a^/ structure coefficient^b^	*p*	Factor loadings^a^/ structure coefficient^b^	Standardized Factor loadings^a^/ structure coefficient^b^	*p*
Substance use behavior	➔	Suicidal behavior	0.53	0.24	0.013	0.34	0.18	<0.001
Substance use behavior	➔	Cigarette use	1	0.44		1	0.91	
Substance use behavior	➔	Alcohol use	0.55	0.40	<0.001	0.35	0.51	0.018
Substance use behavior	➔	MDMA use	0.24	0.35	0.008	0.17	0.28	<0.001
Substance use behavior	➔	Ketamine use	0.24	0.31	0.015	0.18	0.31	<0.001
Suicidal behavior	➔	Suicidal idea	1	0.64		1	0.59	
Suicidal behavior	➔	Suicidal planning	0.95	0.67	<0.001	0.91	0.64	<0.001
Suicidal behavior	➔	Suicidal attempts	0.72	0.66	<0.001	0.94	0.72	<0.001

^a^ coefficients for measurement model; ^b^ coefficient for structure model

## Discussion

The first major finding of the present study, according to the latent variables analysis, was that substance use behavior positively contributes to suicidal behavior in both girls and boys. Substance use may lead to failure in developmental tasks, such as healthy interaction with peers, and in daily obligations, such as attending school or completing school work [38], which may further increase the risk of suicidal behavior. Meanwhile, acute drug intoxication may impair users’ judgment, reduce their inhibition, and decrease their impulsive control, all of which increase the likelihood of suicidal behavior [39]. Chronic substance use may also have a negative impact on adolescent brain development [16, 40]. In addition, adolescents who engage in chronic substance use often exhibit changes in behavioral, affective, and cognitive processes characterized as underdeveloped regulation of aggression and impulsivity [41]. Thus, substance use may contribute to suicidal behavior via multiple pathways.

The present study found that although there were gender differences in substance use and suicidal behavior, the contribution of adolescent substance use to suicidal behavior did not differ between genders. This result was consistent with the results of a previous study that reported that adolescent alcohol use is linked to suicidal behavior, although this association did not differ between boys and girls [42]. Therefore, both results suggest the existence of a gender-independent mechanism underlying the link between substance use behavior and suicidal behavior in adolescents. Further study is needed to explore this mechanism.

The present study found that the rates of all types of suicidal behavior were higher in girls than in boys. A previous study in the US also reported that adolescent girls had higher rates of suicidal ideation, planning and attempts than adolescent boys [43]. The present study found that girls had lower rates of smoking and alcohol use than boys but observed no significant gender differences in the rates of MDMA and ketamine use. However, the results of latent group comparisons in the present study indicated that boys had higher levels of substance use than girls. This result, obtained by latent analysis, provides robust evidence of a gender difference in adolescent substance use because adolescents typically use more than one type of drug [24]. Several factors may be responsible for the gender difference in adolescent substance use behavior. First, adolescent females have lower impulsivity, sensation-seeking and disinhibition than adolescent boys [44]. Second, attention deficient hyperactivity disorder, which may be linked to substance use, is more common in boys than in girls [45]. Third, environmental factors, such as cultural effects, which include cultural variations in sensation-seeking and impulse control, and peer effects, which include attracting friends/acceptance and contacting deviant peers, may also be responsible for the gender difference in substance use [44, 46, 47].

There were several limitations of this study. First, the data were provided by the adolescent subjects; thus, the possible issue of shared-method variance resulting from the sole data source requires careful consideration. Second, depression was not taken into consideration in our study. Depression is a risk factor for suicidal behavior and a common comorbidity for adolescents with substance uses [48]. Third, only adolescents who attended school were enrolled in this research. Fourth, the dichotomous response and lower prevalence rates for selected substance as well as not including other substance such as marmarijuana, cocaine, opioids, may limit the generalization of the findings. Fifth, the causal relationship cannot be determined because of the cross-sectional nature of the study. The relationships between adolescents’ substance use and their suicidal behavior could be influenced by several factors: young people may consider suicide because of substance use, they may use substances to cope with suicidal crises or both substance abuse and suicidal behavior may be the result of various confounders. Further study is therefore needed to determine precise causality, as other relationships may also exist that influence the relationship between substance and suicidal behavior. Despite these limitations, the present study increases our understanding of gender issues on adolescent substance use, as adolescent substance use can manifest as different behavioral phenomena. In addition, the contribution of adolescent substance use to suicidal behavior was found to be gender-independent. This finding suggests that mental health professionals should pay similar attention to substance use in girls and boys when developing and implementing prevention and intervention strategies for suicidal behavior in adolescents.

## Abbreviations

CFI: Comparative fit index
K-SADS-E: Kiddie schedule for affective disorders and schizophrenia
MDMA: 3,4-methylenedioxymethamphetamine
MI: Measurement invariance
Q-ESU: The questionnaire for experience in substance use
RMSEA: Root mean square error of approximation
SEM: Structural equation modeling
